# General self-efficacy modifies the effect of stress on burnout in nurses with different personality types

**DOI:** 10.1186/s12913-018-3478-y

**Published:** 2018-08-29

**Authors:** Yongcheng Yao, Shan Zhao, Xia Gao, Zhen An, Shouying Wang, Hongbin Li, Yuchun Li, Liyun Gao, Lingeng Lu, Ziming Dong

**Affiliations:** 1 School of Public Health, Xinxiang Medical University, Xinxiang, 453003 Henan China; 2 School of Basic Medical Sciences, Zhengzhou University, Zhengzhou, 450001 Henan China; 3 Department of Chronic Disease Epidemiology, Yale School of Public Health, School of Medicine, Yale Cancer Center, Yale University, 60 College Street, New Haven, CT 06520-8034 USA

**Keywords:** Nurse, Job-related burnout, Personality, General self-efficacy, Machine learning, Interaction

## Abstract

**Background:**

Burnout is a health problem in nurses. Individuals with a certain personality are more susceptible to job-related burnout. General self-efficacy (GSE) is an important predictor of job-related burnout. The relationships between general self-efficacy, job-related burnout and different personality types are still not clear. This study aims to analyze the relationships of job-related burnout, stress, general self-efficacy and personality types, as well as their interactions in job-related burnout.

**Method:**

A cross-sectional survey of 860 nurses was conducted between June and July 2015 in China. We measured their job-related burnout using the scale of the Maslach Occupational Burnout Scale, and personality, stress, and GSE. Machine learning of generalized linear model were performed.

**Results:**

Maslach Burnout Inventory (MBI) professional efficacy was significantly associated with gender, marital status, age, job title and length of service. A machine learning algorithm showed that stress was the most important factor in job-related burnout, followed by GSE, personality type (introvert unstable), and job title. Individuals with low GSE and either introversion or unstable (high neuroticism) personality seemed to have stronger burnout when they faced stress (regardless of stress intensity) compared to others.

**Conclusion:**

Stress, GSE and introvert unstable personality are the top three factors of job-related burnout. GSE moderates the effect of stress on burnout in nurses with extroversion or neuroticism personality. Reducing stress, increasing GSE, and more social support may alleviate job-related burnout in nurses. Nurses with introvert unstable personality should be given more social support in reducing stress and enhancing their GSE.

**Electronic supplementary material:**

The online version of this article (10.1186/s12913-018-3478-y) contains supplementary material, which is available to authorized users.

## Background

Burnout is characteristic of depersonalization, emotional exhaustion and low personal accomplishment [1]. Job-related burnout in health care sector not only leads to decreased effectiveness at work, but may also interfere human perception, affecting an individual’s appropriate judgement, reducing the ability to predict accidents, consequently leading to the illegal operations and even the occurrence of medical accidents, deteriorating the quality of care provided to patients [2, 3]. Burnout frequently occurs in people-oriented profession, and especially medical staffs, who provide care services for patients and face more challenges such as the demanding relationships with patients and their relatives, interactions with coworkers in teams, are more susceptible to job-related burnout [4]. A survey of medical staffs showed that more than one-third of the participants (35.8%) reported themselves at the high risk of job-related burnout, 27.2% had a high degree of exhaustion, 10.0% had a certain degree of cynicism, 3.2% lacked professional efficacy [5]. Another survey of 218 medical staffs showed that 42.1% of the subjects had a certain degree of exhaustion, 22.7% had a certain degree of cynicism, 48.6% lacked professional efficacy [6]. One recent study shows over half of nurses working in Veterans Health Administration (VHA) of the Department of Veterans Affairs suffer emotional exhaustion and/or low accomplishment and/or high depersonalization [7]. Margues and colleagues reported that there were 59% and 41% nurses with high level of emotional exhaustion and lack of personal accomplishment, respectively, in a University hospital of Portugal [8]. The prevalence of burnout was 12% in pediatric palliative care provider in the United States [9], whereas its prevalence was relatively high in mental healthy, ranging from 21 to 67% [10–12].

Personality is a sum of psychological characteristics in a relatively stable individual, reflecting one’s adaptability to the environment based on the unique behavior patterns and ways of thinking, and is a product of the interaction with the acquired social environment on the basis of natural qualities. The involvement of personality has been reported in the development of burnout, and some individuals with a certain personality trait are more susceptible to job-related burnout [13, 14]. General characters for the vulnerable individuals include unrealistic ideals and expectations, low self-worth and judgment, lack of self-confidence, lack of accurate understanding of their advantages and limitations [15, 16]. In contrast, those with active coping strategies and the sense of self-efficacy are relatively more immune to burnout [17, 18]. Individuals with a stable extrovert personality have a strong motivation in their actions, while those having cynicism and extroversion appear at the risk of burnout and other mental illnesses [19, 20] . Studies have shown that medical staffs have a stronger emotional response and stubborn behavior compared to the general population [21]. Furthermore, personality types are associated with the sub-dimensions of job-related burnout; exhaustion and cynicism more frequently occur in individuals with the type A personality. Emotional stability is negatively correlated with psychological fatigue, depression, and irritability [22]. A meta-analysis shows that Type A personality was linked to personal accomplishment [23].

General self-efficacy (GSE) refers to an overall self-confidence that an individual responds to different environmental challenges or face new things. It predicts an individual’s behavior, thinking, and emotional reactions. Studies shows that GSE has a significant direct and indirect association with mental health such as depression, anxiety and helplessness [1]. Another study shows that both GSE and professional efficacy are in significantly negative correlation with exhaustion, suggesting that GSE is an important predictor of job-related burnout [24]. Individuals with low GSE also have low self-esteem and pessimistic thoughts of their accomplishments. Job-related burnout can, in turn, reduce self-efficacy, leading to depression, irritability, helplessness, anxiety and other negative emotions [25, 26]. An inverse correlation was found between the self-efficacy and each of the three dimensions of burnout in nurses [27]. Therefore, GSE is an important factor in relation to burnout.

In the previous study, we found that high GSE could not only significantly ameliorate stress but also improve job-satisfaction in nurses [28], both of which are related to burnout and personality. However, it is still unclear how GSE, job-related burnout and different personality types are related to each other in nurses. Burnout is a critical and well-studied outcome of job-stress [29]. High self-efficacy and optimistic personality may protect nurses from the negative effect of job-stress [23, 30]. Thus, stress, GSE and personality may orchestrate together in job-related burnout. Thus, the purposes of this cross-sectional study aimed to investigate the relationships between job-related burnout, stress, GSE and personality type, as well as their interactions in job-related burnout in nurses in China.

## Methods

### Participants

The protocol of this study was approved by the ethics committee of Xinxiang Medical University. This study was conducted through a convenience sample of registered nurses from five municipal hospitals in Henan Province, China. The criteria for the inclusion are those who are registered nurses and have worked in a nurse position for at least 1 year. Based on the criteria, 1100 registered nurses were identified eligible through the human resource department of the hospitals. Of them, 860 agreed to participate when they were informed about the purposes of this study, and successfully completed the survey. The participation rate was 78.2%.

### Study instruments

#### Socio-demographic characteristics

A questionnaire (Additional file 1) was handed to the participants in the hospitals for the collection of selected socio-demographic characteristics including gender, age, professional experience, marital status, hospital department, and job title.

### Maslach burnout inventory – general survey

Burnout syndrome was assessed using the Maslach Burnout Inventory-General Survey (MBI-GS) [31], which was previously translated into Chinese with a good reliability and validity in a Chinese sample [32, 33]. The MBI-GS consists of three dimensions with a total of 15 items rating on a Likert scale from 0 to 6 points: exhaustion (EX, five items), cynicism (CY, four items), and professional efficacy (PE, six items). The score of each dimension is the sum of the items in that dimension. The level of burnout is positively related to the score. Since PE is scored in an opposite direction, the level of professional efficacy is negatively related to PE score. The Cronbach’s Alpha of the scale in this study was 0.850.

The cutoff points were taken to evaluate job-related burnout based on the criteria used by Li Yongxin with minor modification [34]. The cutoff point of upper one two-third of each dimension of the survey sample was used for job-related burnout (EX of 14, CX of 7, PE of 14). The burnout degree of the survey sample was divided into the following four levels: Zero burnout (all three dimensions scoring below the cutoff points); Mild burnout (any one dimension scoring above the cutoff point); Moderate burnout (any 2 dimensions scoring above the cutoff points), and Severe burnout (all three dimensions scoring above the cutoff points).

#### Measurement of general self-efficacy

The Schwazer’s GSE Scale (Chinese version) was applied to measure GSE [35]. It consists of 10 items on a 4-point rating scale. Higher scores suggest higher levels of GSE. Homogeneity reliability scored 0.883 in this study.

#### Measurement of stress

We adopted the Occupational Stress Inventory-Revised (OSI-R) [36] to assess the levels of stress, which consists of 20 items, 10 each for psychological and physical stress. Each item scales 5 points. The sum score was calculated for psychological and physical stress, respectively. A high score indicates a high level of the stress. Its consistency reliability in this study was 0.881.

#### Measurement of personality type

Personality was assessed using two scales of the simplified Chinese version of Eysenck’s Personality Questionnaire-Revised (EPQ-RSC) [37] provides an excellent instrument for personality research, which includes 24 items with each scoring either 0 or 1. The neuroticism scale (EPQ-N), which assesses emotional stability while the extroversion scale (EPQ-E) assesses the need for emotional stimulation. Each scale was divided into high and low categories based on the medians as the cutoff points as reported in literature, an accepted method in analyzing the psychometric scale [38]. A high score was defined as an EPQ-E C60 and an EPQ-N C61 [39], and based on which four types of personality were classified: introvert stable (low EPQ-E, low EPQ-N), extrovert stable (high EPQ-E, low EPQ-N), extrovert unstable (high EPQ-E, high EPQ-N) and introvert unstable (low EPQ-E, high EPQ-N).

### Data analysis

Data were recorded and analyzed using Epidata3.1 and SPSS (version 15 for Windows). Numerical variables are presented as mean ± standard deviation (SD). A two-tailed test yielding *p* < 0.05 was considered statistically significant. Either t-test or Analysis of Variance (ANOVA) was used to analyze the differences between the groups, and post hoc Bonferroni tests were performed to verify the differences between the specific groups in analyzing associations of demographic variables with job-related burnout syndrome components, and general self-efficacy. Machine learning of generalized linear algorithm (GLM) using H2O.ai (http://docs.h2o.ai/h2o/latest-stable/h2o-docs/flow.html) was performed to analyze the importance of the factors in job-related burnout, in which the subjects were randomly (ratio = 0.70) divided into training and validation datasets [40]. GLM was fitted to estimate the set of parameters by maximizing the log-likelihood of the data for the best model. A Gaussian family and 10-fold cross-validation were set, and the default was set for other parameters in the modeling. ModGraph [41] was used to construct graphs for the interactions between GSE, stress and personality in job-related burnout after multivariate regression analyses for their interactions following the recommendation by Dawson and Richter [42] to analyze the 3-way interactions in either neuroticism or extroversion personality, respectively, to increase the analysis power by keeping the number of participants not too small in each subgroup.

## Results

### The prevalence of job-related burnout in nurses

Of all the 860 nurses who agreed to participate and successfully completed the survey, 68.1% (*n* = 586) of the participants had job-related burnout. Of 586 nurses, 279 (32.4%) were mild job-related burnout, 238 (27.7%) were moderate job-related burnout, 69 (8.0%) were severe job-related burnout.

### Associations of demographic variables and personality types with burnout syndrome components, and general self-efficacy of nurses

Association analytic results are shown in Table 1. There was statistically significant association between gender and MBI-GS (*p* < 0.05) with males having higher job-related burnout than females. Male nurses showed significantly higher professional efficacy than female ones (*P* < 0.01). There was significantly higher MBI-GS professional efficacy in single nurses compared with married nurses (*P* < 0.05). Professional efficacy (PE) reduced gradually with both age and the length of service in years (*P* < 0.01). Post hoc test results showed that nurses ageing 30~ years or with 1~ years of service scored significantly higher on the professional efficacy subscale than nurses 40~ years or with 20~ years, respectively. There was a significant association between the exhaustion scores and department (*P* < 0.05); the nurses in emergency departments scored significantly higher on the exhaustion subscale than nurses in Obstetrics and gynecology and other departments. Nurses with a primary title had significantly higher professional efficacy scores than those with an intermediate title (*P* < 0.05).

**Table 1:** Associations of Demographic variables with job-related burnout syndrome components, and General Self-efficacy in nurses (mean ± SD)

Variable	N	MBI-GS	MBI-GS: EX	MBI-GS: CY	MBI-GS: PE	GSE
Gender						
Male	48	35.2 ± 13.5	11.9 ± 6.9	8.4 ± 6.2	14.9 ± 8.1	24.9 ± 5.4
Female	812	30.9 ± 14.1	13.2 ± 6.7	7.0 ± 5.4	10.7 ± 8.8	25.5 ± 5.6
t		2.057^*^	−1.268	1.682	3.241^**^	−0.688
Marital status						
Single	448	31.4 ± 14.2	12.7 ± 6.6	7.1 ± 5.4	11.6 ± 8.5	25.3 ± 5.5
Married	412	30.8 ± 14.0	13.5 ± 6.8	7.1 ± 5.5	10.2 ± 9.0	25.6 ± 5.7
t		0.678	−1.820	0.096	2.424^*^	−0.749
Age						
< 30	622	31.4 ± 13.7	12.8 ± 6.6	7.0 ± 5.3	11.5 ± 8.7	25.2 ± 5.4
30~	168	31.7 ± 15.6	14.2 ± 7.0	7.6 ± 5.9	10.0 ± 8.7	25.5 ± 6.2
40~	70	27.5 ± 13.3	12.8 ± 6.7	6.4 ± 5.8	8.3 ± 9.3	27.5 ± 5.3
F		2.616	2.649	1.362	5.484^**^	5.427^**^
Length of service (yrs)						
1~	666	31.4 ± 13.9	12.8 ± 6.6	7.0 ± 5.3	11.5 ± 8.7	25.2 ± 5.5
10~	116	31.7 ± 15.8	14.5 ± 7.0	7.8 ± 6.0	9.3 ± 8.3	25.6 ± 5.8
20~	78	28.0 ± 13.2	13.2 ± 6.9	6.5 ± 5.9	8.4 ± 9.1	27.0 ± 6.1
F		2.078	2.921	1.675	6.855^**^	3.621^*^
Department						
Emergency	114	34.7 ± 14.0	15.1 ± 7.1	8.0 ± 5.3	11.6 ± 8.3	24.9 ± 5.5
Surgical	145	31.5 ± 14.9	13.6 ± 6.9	7.6 ± 5.6	10.3 ± 8.4	25.0 ± 5.4
Pediatric	44	32.8 ± 15.5	14.8 ± 7.9	7.0 ± 6.1	11.0 ± 9.7	25.9 ± 6.1
Obstetrics and gynecology	95	29.9 ± 13.2	12.0 ± 5.9	7.0 ± 4.7	10.9 ± 8.9	26.1 ± 6.2
Medicine	186	29.7 ± 13.9	13.1 ± 6.9	6.4 ± 5.4	10.3 ± 8.7	25.3 ± 5.4
Mental Health	100	33.2 ± 13.1	12.4 ± 6.6	8.0 ± 5.7	12.8 ± 9.1	24.9 ± 5.7
Other	176	29.0 ± 13.8	12.0 ± 6.0	6.3 ± 5.3	10.7 ± 9.0	25.9 ± 5.6
F		2.810^*^	3.764^**^	2.198^*^	1.224	1.029
Job title						
Primary	718	31.3 ± 14.1	13.0 ± 6.7	7.0 ± 5.3	11.2 ± 8.7	25.1 ± 5.5
Intermediate	142	30.4 ± 14.2	13.5 ± 7.0	7.4 ± 6.0	9.5 ± 8.9	26.8 ± 6.0
t		0.707	−0.827	−0.596	2.173^*^	−3.260^**^
Personality type						
Introvert Stable	170	28.3 ± 13.0	11.6 ± 6.3	5.7 ± 4.3	10.9 ± 9.2	25.8 ± 5.2
Extrovert Stable	223	24.9 ± 12.7	10.7 ± 5.9	5.2 ± 5.1	8.9 ± 8.2	27.6 ± 5.4
Introvert Unstable	283	36.2 ± 14.1	15.0 ± 6.7	9.0 ± 5.8	12.2 ± 8.6	23.3 ± 5.0
Extrovert Unstable	184	33.5 ± 13.2	14.4 ± 6.8	7.5 ± 5.2	11.6 ± 8.9	25.7 ± 5.9
F		34.29^***^	23.07^***^	26.80^***^	6.38^***^	27.93^***^

Note: ^*^P < 0.05, ^**^P < 0.01, ^***^P < 0.001. *MBI-GS* Maslach burnout inventory-general survey, *EX* Exhaustion, *CY* Cynicism, *PE* Professional efficacy, *GSE* General self-efficacy

There was no significant relationships between either the gender or marital status and GSE (*p* > 0.05). GSE increased gradually with either age or the length of service increasing. Post hoc test results showed that nurses ageing 40~ years scored significantly higher on the GSE subscale than other age groups (*P* < 0.05), and nurses with 20~ years of service scored significantly higher on the GSE subscale than nurses with 1~ years (*P* < 0.05). Nurses with an intermediate titles had significantly higher GSE scores than those with a primary title (entry level) (*P* < 0.01). There was no significant relationship between different departments and GSE (*p* > 0.05).

Differences in all dimensions of job-related burnout and GSE were statistically significant in the four personality types (*P* < 0.001) (Table 1) Post hoc test results showed that stable nurses scored significantly lower on MBI-GS and the exhaustion subscale than unstable ones (P < 0.01). Extrovert stable nurses scored significantly lower professional efficacy than unstable one (P < 0.05).

### Importance of risk factors in job-related burnout

**Fig. 1:**
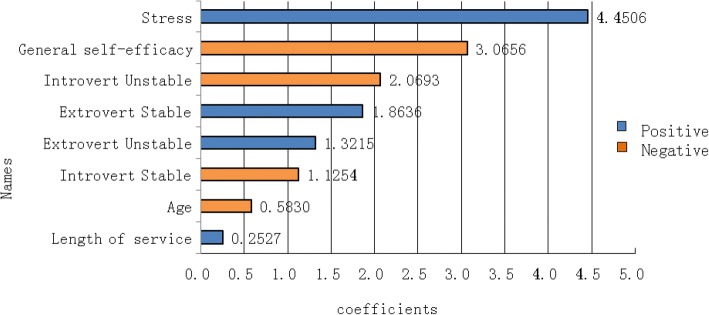
The variable importance in job-related burnout. The factors are ranked based on their importance in burnout. The blue bar stands for the factor having a positive coefficient with burnout, while the orange bar stands for the factor having a negative coefficient with burnout

Several risk factors of burnout, such as gender, age, marital status, work shift and personality, have been identified in nurses [43, 44]. However, the relative importance of these factors in burnout has not been reported. To explore the importance of the risk factors in job-related burnout, we performed H2O’s machine learning (H2OFlow of H2O.ai) of generalized linear model algorithm, and the results are shown in Fig. 1 (blue stands for a positive association, and orange for a negative one). Stress ranked no. 1 (standardized coefficient = 4.89) in the risk factors of job-related burnout, followed by GSE (standardized coefficient = − 3.39), introvert unstable personality, job title, extrovert and introvert stable personality, age, marital status, extrovert unstable personality, gender and length of service. The risk factors of stress, introvert and extrovert unstable personality, job title, and length of service showed positive correlations with job-related burnout. In contrast, GSE, extrovert and introvert stable personality, age, marital status and gender (women) had negative correlation with job-related burnout.

### Joint effect of GSE, burnout and personality types on stress

Table 2 shows the correlations between variables. There was a significantly positive and moderate correlation between burnout and stresses and Extroversion (*r* = 0.44, 0.35 respectively, *p* < 0.01). Burnout was significantly negatively correlated with GSE and Neuroticism (*r* = − 0.39, 0.23 respectively, *p* < 0.01).

**Table 2:** Pearson Correlations of the variables (*n* = 847)

Variable	*M*	*SD*	Burnout	GSE	Stress	Extroversion
Burnout	30.8	13.8	1.00			
GSE	25.4	5.5	−0.38^**^	1.00		
Stress	52.7	11.6	0.43^**^	−0.29^**^	1.00	
Extroversion	7.1	2.7	−0.23^**^	0.27^**^	−0.31^**^	1.00
Neuroticism	5.9	3.0	0.35^**^	−0.28^**^	0.50^**^	−0.25^**^

Note: ^*^p < 0.05, ^**^p < 0.01. *GSE* General self-efficacy

Table 3 shows the results of three different multivariate models. In the model 1, as expected, stress, and GSE but extroversion personality showed significant associations with burnout, with stress having a positive association and GSE having a negative association. In the model 2 of the 2-way interactions, there was a significant interaction between stress and extroversion, but neither between GSE and extroversion, nor between GSE and stress. However, in the model 3 of the 3-way interactions, the interaction of stress, GSE and extroversion was significant (*p* = 0.039), whereas the significance of main effect of stress was not held, and GSE still remained significant.

**Table 3:** Effects of GSE, extroversion and stress on burnout in multivariate regression analyses

Variable	Model 1		Model 2		Model 3	
	β	*p* value	β	*P* value	β	*P* value
stress	0.403	< 0.001	0.391	0.039	−0.493	> 0.05
GSE	−0.669	< 0.001	−1.21	0.01	−3.124	0.003
E	−0.25	> 0.05	1.707	> 0.05	−4.988	> 0.05
stress×GSE			0.011	> 0.05	0.046	0.013
stress×E			−0.037	0.005	0.089	> 0.05
GSE × E			0	> 0.05	0.261	0.044
stress×GSE × E					−0.005	0.039
Adjusted R2	0.256		0.261		0.264	
ΔR2	0.258	< 0.001	0.008	< 0.05	0.004	0.039

β: standardized coefficient; *GSE* General self-efficacy, *E* Extroversion

**Fig. 2:**
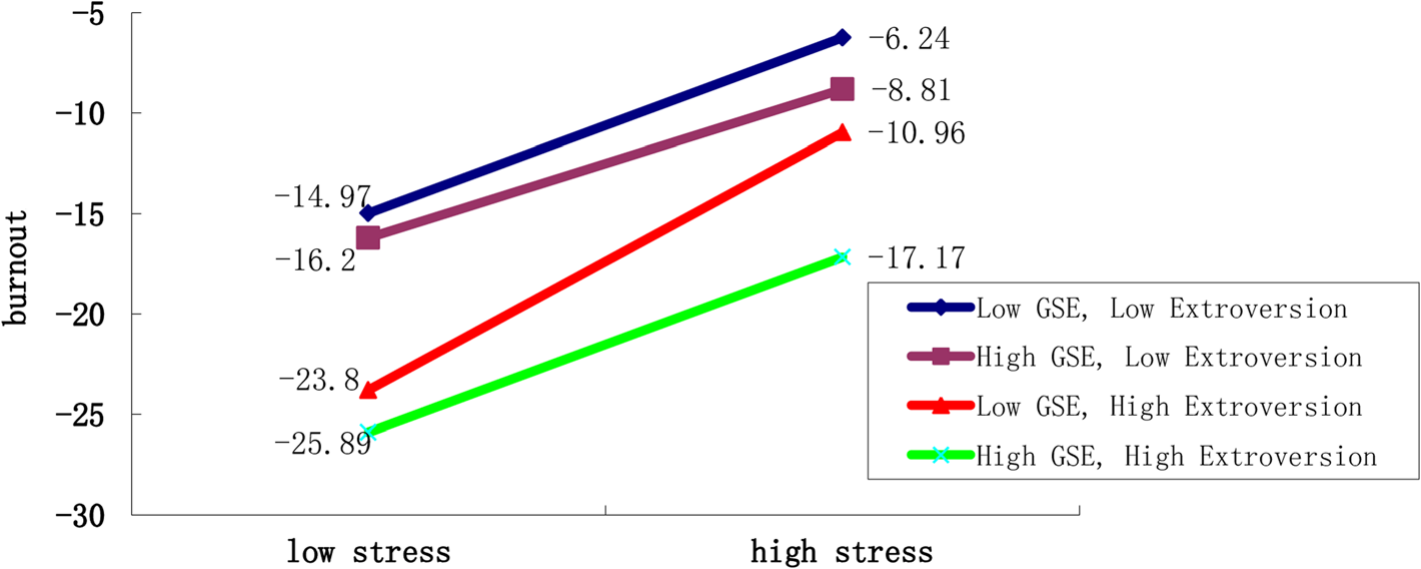
Interaction of GSE, stress, extroversion personality on burnout in nurses

To visualize the effect of stress, GSE and extroversion in burnout, we further constructed the graph based on the method as described by Dawson and Richter [42] (Fig. 2). Individuals with low GSE and low extroversion seemed to have stronger burnout when they experienced stress (regardless of stress intensity) compared to others. The introvert individuals had an overall higher burnout than those extrovert regardless of the GSE.

**Fig. 3:**
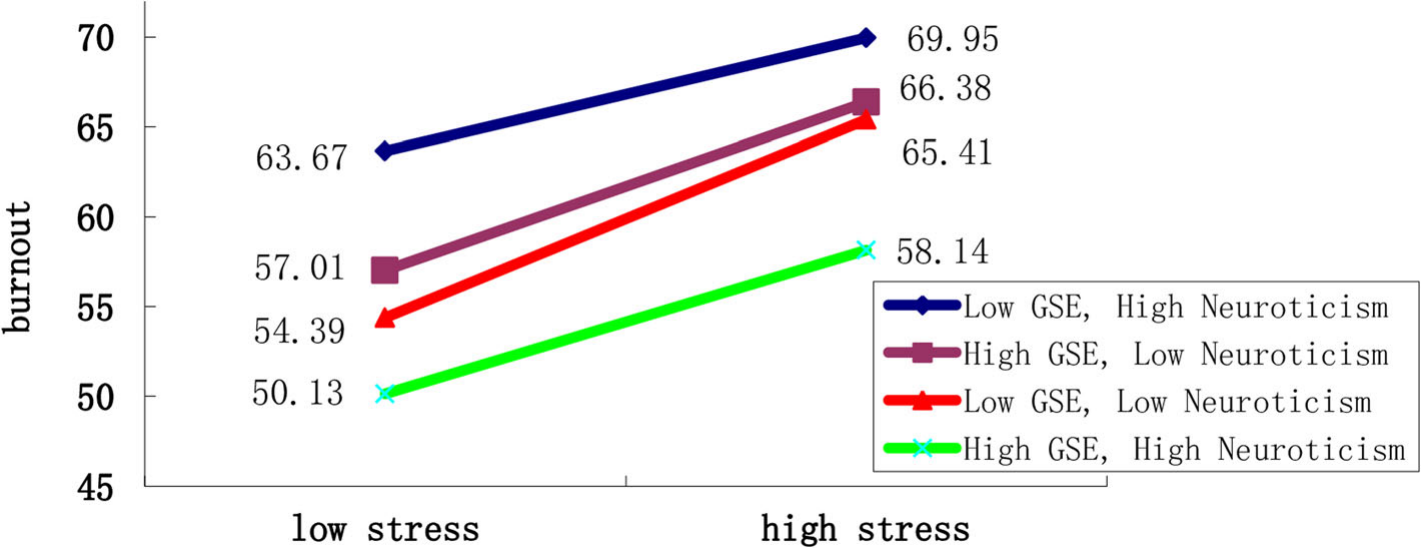
Interaction of GSE, stress and neuroticism personality on burnout in nurses

Similarly, we examined the interaction of stress, GSE and neuroticism personality in burnout (Table 4). In the model 3, we found there was a significant 3-way interaction of stress, GSE and neuroticism personality (*p* = 0.045). Figure 3 shows that individuals with low GSE and unstable (high neuroticism) seemed to have stronger burnout when they faced stress (regardless of stress intensity) compared to others, while those with high GSE and unstable had the lowest burnout.

**Table 4:** Effects of GSE, neuroticism and stress on burnout in multivariate regression analyses

Variable	Model 1		Model 2		Model 3	
	β	*P* value	β	*P* value	β	*P* value
stress	0.348	< 0.001	0.17	> 0.05	0.809	0.038
GSE	−0.645	< 0.001	−0.979	0.005	0.181	> 0.05
N	0.587	< 0.001	0.913	> 0.05	6.032	0.029
stress×GSE			0.007	> 0.05	−0.017	> 0.05
stress×N			−0.003	> 0.05	−0.102	0.046
GSE × N			−0.007	> 0.05	−0.201	0.047
stress×GSE × N					0.004	0.045
Adjusted R2	0.266		0.264		0.267	
ΔR2	0.268	< 0.001	0.001	> 0.05	0.003	0.045

β: standardized coefficient; *GSE* General self-efficacy, *N* Neuroticism

## Discussion

This study showed that the prevalence of job-related burnout in Chinese nurses was 68.1%, and was higher than that reported by Wang [45, 46]. The prevalence of job-related burnout in female was lower than male, and was the highest in nurses who are married, or over 30 years old age, or had over 10 years length of service, or worked in surgical department. This finding is in consistence with the results of our previous studies [44]. This finding also suggests that the job-related burnout in Chinese nurses is a big health problem and should not be ignored. Nurses often directly interact with patients and their families, requiring not only strong medical skills, but also the strong interpersonal relationship skills. Relatively severe pressure and the often duty-shift for nurses make them susceptible to job-related burnout.

Influenced by Chinese traditional culture, women nurses are still predominant in Chinese hospitals, and a very few men would like to take nursing job as their professional career until today. Thus, the lack of the recognition of the nurse profession may be one of the reasons for the sense of low accomplishment in male nurses. Similarly, the sense of low achievement was also observed in younger and unmarried nurses. This finding is consistent with the results of other previous studies [47, 48]. Maslach et al. reported that the levels of job-related burnout in young employees was relatively high [29] . One possibility for young employees is due to the short service, heavy community pressure, poor job adaptability, and the lack of autonomy. With age increasing and job status rising, the job initiative and accomplishment were much stronger and consequently, job-related burnout reduced gradually.

Emergency department is often on duty 24 h a day and 7 days a week to face emergencies and deal with critical patients. In addition, task of nurses in emergency department is heavy with great responsibility, and they are always in highly tense condition. Thus, the job-related burnout in this group is prominent. However, this hospital department-based analysis may not reflect the staff shortages or the volume of patients nurses cared for. Thus, further analyses with the adjustment of the volume of patients and the number of nurses in each department could be performed in the future research.

Self-efficacy refers to the belief that an individual has an ability to take action and achieve a given goal [49]. GSE has nothing to do with the actual skills of individuals, but is related to the self-judgment for the individuals’ decision to apply their skills. It has been reported that individuals with high self-efficacy tend to adopt positive coping strategies when they face with severe pressure, believing that they are able to complete the tasks and do not feel too much pressure [50]. Studies have shown that personality type is an important factor in job-related burnout, and individuals with a certain personality type tend to be susceptible to job-related burnout [51]. In our study, the interactions between GSE, stress and extroversion or neuroticism showed that Extrovert nurses with better GSE had less burnout, and unstable ones with low GSE had relatively stronger burnout when they faced job challenges or stress. In the nurses with extroversion but not neuroticism personality, GSE could completely moderate the effect of stress on burnout. Generally, response of emotionally stable nurses to job stress is gentle, and they are quickly calmed down when they face the provocation of the patients or their family members. However, emotional unstable individuals are impulsive, irritable and sensitive, particularly if their GSE are also relatively low, their feelings of burnout are stronger. Thus, this finding suggests that appropriate measures may reduce the effect of stress on burnout based on the personality type of extroversion or neuroticism.

A variety of factors influence job-related burnout. We found that stress is the most important risk factor in job-related burnout, whereas GSE is the most important protective factor. Different personalities had different associations with job-related burnout; introvert unstable is a risk factor, whereas extrovert stable protects individuals from job-related burnout. Due to the limitation of time and data, the study participants enrolled in this study were mainly from the municipal hospitals with relatively heavy patient loads, and may have selection bias leading to overestimate the prevalence of burnout. However, this is a real situation in China, more patients particularly with relatively complicated or severe diseases directly see doctors in a municipal hospital rather than in a primary one. Thus, more systematic and comprehensive research needs to be further conducted by expanding the study to include nurses in primary hospitals where patients are crowded too. However, the advantages of this study include such as a high response rate, a relatively large sample size, and that we revised and improved the threshold value in the evaluation criteria job-related burnout, which was defined by Zhu and colleagues [32, 33]. The revised evaluation criteria take the three factors of job-related burnout into account, not only facilitating a more comprehensive investigation of the situation of job-related burnout, but also helping to take measures targeting prevention and intervention. In addition, we applied machine learning in the evaluation of risk factor importance in job-related burnout, which provides a direction for health policy makers to make strategies to prevent job-related burnout, for example, reducing stress, enhancing GSE, and promoting communications between individuals. Conversely, these strategies such as enhancing GSE, psychiatric help and social support, interpersonal communications may help nurses to reduce burnout when they face job challenges or stress.

## Conclusion

This finding suggests that the job-related burnout in Chinese nurses is a big health problem and should not be ignored. Stress is the most important risk factor, and GSE is the most important protective factor in job-related burnout. Men and nurses in emergency department are susceptible to job-related burnout. The nurses who are more outgoing, have the high self-efficacy, and are married are not susceptible to have job-related burnout, and those with low GSE and unstable introversion personality feel stronger burnout when they face stress. To our knowledge, this is the first study to demonstrate that stress is the most important risk factor of burnout using a machine learning algorithm, and that GSE moderates the effect of stress on burnout in introvert or neuroticism personality in nurses with a relatively large sample size and high response rate in middle China. Thus, a more comprehensive strategy such as enhancing individuals’ general self-efficacy, psychiatric help and social support should be exercised in hospital, and may help nurses with introvert unstable personality to reduce burnout when they face job challenges or stress.

## Additional file

Additional file 1: Supplementary questionnaire. Occupational health questionnaire of burnout, general self-efficacy, personality and stress used in this study. (DOC 143 kb)

## Abbreviations

CY: Cynicism
EPQ-E: The extroversion scale
EPQ-N: The neuroticism scale
EPQ-RSC: Eysenck’s Personality Questionnaire-Revised
EX: Exhaustion
GSE: General self-efficacy
MBI-GS: Maslach Occupational Burnout Scale
OSI-R: Occupational Stress Inventory-Revised
PE: Professional efficacy
