# Supplementary material for: General self-efficacy modifies the effect of stress on burnout in nurses with different personality types
Source: BMC Health Serv Res. 2018 Aug 29;18:667. doi: 10.1186/s12913-018-3478-y (PMC6114188; doi:10.1186/s12913-018-3478-y)
Supplement: Supplementary file 1 — Supplementary questionnaire. Occupational health questionnaire of burnout, general self-efficacy, personality and stress used in this study. (DOC 143 kb) [file 12913_2018_3478_MOESM1_ESM.doc]

**Occupational health questionnaire**

**Dear friend, the purpose of this survey is to study the impact of occupation environment on the health of employees. The information does not involve personal privacy issues. Please select the best choice from the options in this table according to your work and health status. Your answers will provide useful references for improving your working conditions and promoting physical and mental health in the future. Please answer every question carefully. The protocol of this study was approved by the ethics committee of Xinxiang Medical University. If you don't like it, you can refuse to answer. Thank you very much!**

**Demographic variables：**

**1. Gender：** ①Male ② Female

2. **Age**

**3. Length of service (yrs)**___ ____

**4. Marital status** ①Single ②Married

**5. Department：** ①Emergency ②Surgical ③Pediatric ④Obstetrics and gynecology

⑤Medicine ⑥Mental Health ⑦Other

**6. Job title：**①Primary ②Intermediate

**1. Maslach Burnout Inventory General Survey**

**Please tick the appropriate number according to your own experience to determine how often they occur on you.**

| **0** | **1** | **2** | **3** | **4** | **5** | | | | | | **6** | | |
| --- | --- | --- | --- | --- | --- | --- | --- | --- | --- | --- | --- | --- | --- |
| **Never** | **very seldom** | **seldom** | **sometimes** | **frequently** | **almost always** | | | | | | **always** | | |
| **1. I feel emotionally drained from my work** | | | | | | **0** | **1** | **2** | **3** | **4** | | **5** | **6** |
| **2. I fell used up at the end of the workday** | | | | | | **0** | **1** | **2** | **3** | **4** | | **5** | **6** |
| **3. I feel fatigued when I get up in the morning and have to face another day on the job** | | | | | | **0** | **1** | **2** | **3** | **4** | | **5** | **6** |
| **4. Working with people all day is really a strain for me** | | | | | | **0** | **1** | **2** | **3** | **4** | | **5** | **6** |
| **5. I feel burned out from my work** | | | | | | **0** | **1** | **2** | **3** | **4** | | **5** | **6** |
| **6. I have become more callous toward people since I took this job** | | | | | | **0** | **1** | **2** | **3** | **4** | | **5** | **6** |
| **7. I worry that this job is hardening me emotionally** | | | | | | **0** | **1** | **2** | **3** | **4** | | **5** | **6** |
| **8. I feel frustrated by my job** | | | | | | **0** | **1** | **2** | **3** | **4** | | **5** | **6** |
| **9. I do not really care what happens to some recipients** | | | | | | **0** | **1** | **2** | **3** | **4** | | **5** | **6** |
| **10. I deal very effectively with the problems of my recipients** | | | | | | **0** | **1** | **2** | **3** | **4** | | **5** | **6** |
| **11. I feel I am positively influencing other people’s lives through my work** | | | | | | **0** | **1** | **2** | **3** | **4** | | **5** | **6** |
| **12. I can easily create a relaxed atmosphere with my recipients** | | | | | | **0** | **1** | **2** | **3** | **4** | | **5** | **6** |
| **13. I feel exhilarated after working closely with my recipients** | | | | | | **0** | **1** | **2** | **3** | **4** | | **5** | **6** |
| **14. I have accomplished many worthwhile things in this job** | | | | | | **0** | **1** | **2** | **3** | **4** | | **5** | **6** |
| **15. In my work, I deal with emotional problems very calmly** | | | | | | **0** | **1** | **2** | **3** | **4** | | **5** | **6** |

**2. General Self-efficacy**

**Please judge how often they occur on you based on your feelings and experiences, and tick the appropriate number.**

**①Never true ②sometimes true ③almost always true ④**always true

| **1. I can always manage to solve difficult problems if I try hard enough** | **1** | **2** | **3** | **4** |
| --- | --- | --- | --- | --- |
| **2. If someone opposes me, I can find the means and ways to get what I want** | **1** | **2** | **3** | **4** |
| **3. It is easy for me to stick to my aims and accomplish my goals** | **1** | **2** | **3** | **4** |
| **4. I am confident that I could deal efficiently with unexpected events** | **1** | **2** | **3** | **4** |
| **5. Thanks to my resourcefulness, I know how to handle unforeseen situations** | **1** | **2** | **3** | **4** |
| **6. I can solve most problems if I invest the necessary effort** | **1** | **2** | **3** | **4** |
| **7. I can remain calm when facing difficulties because I can rely on my coping abilities** | **1** | **2** | **3** | **4** |
| **8. When I am confronted with a problem, I can usually find several solutions** | **1** | **2** | **3** | **4** |
| **9. If I am in trouble, I can usually think of a solution** | **1** | **2** | **3** | **4** |
| **10. I can usually handle whatever comes my way** | **1** | **2** | **3** | **4** |

**3.Eysenck’s Personality Questionnaire-Revised (EPQ-RSC)**

**Please answer the following questions according to your actual situation. Only draw a square root on the right answer. You have to answer every question**

| **1. Does your mood often go up and down?** | Yes | No |
| --- | --- | --- |
| **2. Would it upset you a lot to see a child or an animal suffer?** | Yes | No |
| **3. Are you a talkative person?** | Yes | No |
| **4. If you say you will do something, do you always keep your promise no matter how inconvenient it might be?** | Yes | No |
| **5. Do you ever feel ‘just miserable’ for no reason?** | Yes | No |
| **6. Would being in debt worry you?** | Yes | No |
| **7. Are you rather lively?** | Yes | No |
| **8. Were you ever greedy by helping yourself to more than your share of anything?** | Yes | No |
| **9． Are you an irritable person?** | Yes | No |
| **10. Would you take drugs which may have strange or dangerous effects?** | Yes | No |
| **11. Do you enjoy meeting new people?** | Yes | No |
| **12. Have you ever blamed someone for doing something you knew was really your fault?** | Yes | No |
| **13. Are your feeling easily hurt?** | Yes | No |
| **14. Do you prefer to go your own way rather than act by the rules?** | Yes | No |
| **15. Can you usually let yourself go and enjoy yourself at a lively party?** | Yes | No |
| **16. Are all your habits good and desirable one?** | Yes | No |
| **17. Do you often feel “fed-up”?** | Yes | No |
| **18. Do good manners and cleanliness matter much to you?** | Yes | No |
| **19. Do you usually take the initiative in making new friends?** | Yes | No |
| **20.** **Do you ever curse casually？** | Yes | No |
| **21. Would you call yourself a nervous person?** | Yes | No |
| **22. Do you think marriage is old-fashioned and should be done away with?** | Yes | No |
| **23. Can you easily get some life into a rather dull party?** | Yes | No |
| **24. Have you ever broken or lost something belonging to someone else?** | Yes | No |
| **25. Are you a worrier?** | Yes | No |
| **26. Do you enjoy co-operating with others?** | Yes | No |
| **27. Do you tend to keep in the background on social occasions?** | Yes | No |
| **28. Does it worry you if you know there are mistakes in your work?** | Yes | No |
| **29. Have you ever said anything bad or nasty about anyone?** | Yes | No |
| **30. Would you call yourself tense or “highly strung”?** | Yes | No |
| **31. Do you think people spend too much time safeguarding their future with saving and insurances?** | Yes | No |
| **32. Do you like mixing with people?** | Yes | No |
| **33. As a child were you ever cheeky to your parents?** | Yes | No |
| **34. Do you worry too long after an embarrassing experience?** | Yes | No |
| **35. Do you try not to be rude to people?** | Yes | No |
| **36. Do you like plenty of bustle and excitement around you?** | Yes | No |
| **37. Have you ever cheated at a game?** | Yes | No |
| **38. Do you suffer from “nerves”?** | Yes | No |
| **39. Would you like other people to be afraid of you?** | Yes | No |
| **40. Have you ever taken advantage of someone?** | Yes | No |
| **41. Are you mostly quiet when you are with other people?** | Yes | No |
| **42. Do you often feel lonely?** | Yes | No |
| **43. Is it better to follow society’s rules than go your own way?** | Yes | No |
| **44. Do other people think of you as being very lively?** | Yes | No |
| **45. Do you always practice what you preach?** | Yes | No |
| **46. Are you often troubled about feelings of guilty?** | Yes | No |
| **47. Do you sometimes put off until tomorrow what you ought to do today?** | Yes | No |
| **48. Can you get a party going?** | Yes | No |

1. **Occupational Stress Inventory-Revised（OSI-R）**

**Please select the best choice from the options in this table according to the health condition of the past three months,. Each question has five choices. Please tick the number that best reflects your situation.**

①**Never** ②**seldom** ③**sometimes** ④**frequently** ⑤**always**

| 1． Recently, I feel irritable | ① ② ③ ④ ⑤ |
| --- | --- |
| 2． Recently, I feel depressed | ① ② ③ ④ ⑤ |
| 3． Recently, I feel anxious | ① ② ③ ④ ⑤ |
| 4． Recently, I feel happy | ① ② ③ ④ ⑤ |
| 5．When I go to bed at night, I am often troubled by some thoughts and it is difficult to fall asleep | ① ② ③ ④ ⑤ |
| 6．Recently, I feel poor to cope with difficulties | ① ② ③ ④ ⑤ |
| 7．I found myself complaining about some small things | ① ② ③ ④ ⑤ |
| 8．Recently, I feel upset | ① ② ③ ④ ⑤ |
| 9．I have a sense of humor | ① ② ③ ④ ⑤ |
| 10．I found that everything I do is going smoothly | ① ② ③ ④ ⑤ |
| 11．I gain weight outside of the plan | ① ② ③ ④ ⑤ |
| 12．I have irregular eating habits | ① ② ③ ④ ⑤ |
| 13．Recently, I drank a lot | ① ② ③ ④ ⑤ |
| 14．Recently, I feel burned out | ① ② ③ ④ ⑤ |
| 15．I feel nervous | ① ② ③ ④ ⑤ |
| 16．I have trouble falling asleep and sleeping at night | ① ② ③ ④ ⑤ |
| 17．I feel some indescribable pain | ① ② ③ ④ ⑤ |
| 18．I have some unhygienic food | ① ② ③ ④ ⑤ |
| 19．I feel good | ① ② ③ ④ ⑤ |
| 20．Recently, I feel energetic | ① ② ③ ④ ⑤ |
